# Impact of semaglutide on weight and functional outcomes among obese heart failure patients: a propensity scores matching analysis

**DOI:** 10.1186/s12872-024-04275-2

**Published:** 2024-10-26

**Authors:** Mahmoud Balata, Marc Ulrich Becher

**Affiliations:** 1 University Hospital Bonn, Venusberg-Campus 1, 53127 Bonn, Germany; 2 Department of Internal Medicine and Cardiology, City hospital Solingen, Gotenstraße 1, 42653 Solingen, Germany

**Keywords:** Semaglutide, Glucagon-like peptide 1 receptor agonist, Weight reduction, Heart failure

## Abstract

**Background & objectives:**

Obesity is a common comorbidity in heart failure, yet effective pharmacological options for weight loss in these patients are limited. Semaglutide, a glucagon-like peptide 1 receptor agonist, has shown promise for weight reduction in obese adults. This study aims to evaluate semaglutide’s impact on weight loss, functional status, and clinical outcomes in obese patients with heart failure.

**Methods:**

A retrospective analysis was conducted on all consecutive obese (BMI > 30 kg/m²) patients with heart failure at the University Hospital Bonn outpatient clinic from July 2019 to July 2022. Propensity score matching paired patients receiving semaglutide as an add-on therapy (SEMA) with those on medical therapy alone (Control).

**Results:**

Among 1,942 patients with heart failure screened, 26 matched pairs were identified. At one year, the SEMA group exhibited significant weight loss, with a mean BMI reduction of -2.91 kg/m² (95% CI: -4.27 to -1.55; *p* < 0.001), while the control group showed a non-significant mean change of -0.41 kg/m² (95% CI: -1.08 to 0.26; *p* = 0.22). The difference in BMI between the two groups was statistically significant (mean difference: 3.42 kg/m², 95% CI: 1.43 to 5.42; *p* = 0.001). Improvements by at least one NYHA class were observed in 65% of the SEMA group (*p* < 0.001) compared to 15% of the control group (*p* = 0.18). The SEMA group also showed a significant increase in 6-minute walk distance (6MWD), with a mean difference of 75 m between the groups at one year (95% CI: 0.53 to 150.02; *p* = 0.049). NT-proBNP levels significantly decreased in the SEMA group (*p* < 0.001) compared to the control group (*p* = 0.78), with a statistically significant difference in NT-proBNP between the groups (*p* = 0.048). Both improvements in 6MWD and reductions in NT-proBNP were significantly correlated with BMI percentage reductions.

**Conclusions:**

Semaglutide was associated with significant weight reduction in obese patients with heart failure, accompanied by improved NYHA classification and 6-minute walk distance. Larger, multi-center trials and prospective, randomized controlled trials are warranted. These studies should focus on assessing long-term outcomes, optimizing dosage, and exploring the potential cardiovascular benefits beyond weight reduction.

**Supplementary Information:**

The online version contains supplementary material available at 10.1186/s12872-024-04275-2.

## Introduction

Heart failure stands as a leading cause of morbidity and mortality, with its prevalence steadily increasing [1]. Up to 45% of patients with heart failure struggle with obesity, increasing their susceptibility to hypertension, diabetes, dyslipidemia, myocardial infarction, and worsening heart failure [2–5]. Recognizing these risks, the American Heart Association (AHA) and the European Society of Cardiology (ESC) both recommend obesity management in heart failure patients [6, 7]. Yet, there is a critical gap in safe, effective pharmacological options for weight loss in this population. Moreover, many heart failure patients experience exertional dyspnea, limiting their ability to perform strenuous exercises required for weight loss [8]. Compounding the complexity of managing obesity in heart failure is the “obesity paradox,” where higher BMI patients often show better survival outcomes than their leaner counterparts [9]. This paradox raises questions about the role of intentional weight loss in heart failure management, particularly regarding its impact on clinical outcomes and overall survival.

Semaglutide (Novo Nordisk, Denmark) is a glucagon-like peptide 1 receptor (GLP-1R) agonist initially designed to treat type 2 diabetes [10]. By slowing gastric emptying, suppressing appetite, and enhancing insulin secretion from pancreatic beta cells, semaglutide has been shown to be associated with significant weight loss in obese adults [11]. However, the effects of GLP-1R agonists in heart failure remain controversial. In the FIGHT and LIVE trials, GLP-1R agonists did not lead to significant improvements in left ventricular systolic function, heart failure rehospitalizations, or overall survival [12, 13]. Additionally, they were linked to more serious cardiac events [12]. These findings contrast with more favorable results from trials such as STEP-HFPEF and SELECT [14, 15].

Given the discrepancies in previous studies, this research aims to use real-world data to evaluate the effects of semaglutide on weight loss, functional status, and clinical outcomes in obese patients with heart failure.

## Methods

### Study population

We performed a retrospective analysis of all consecutive obese patients (BMI > 30 kg/m²) with heart failure treated at the outpatient clinic of the University Hospital Bonn between July 2019 and July 2022. Patients with heart failure were identified using ICD codes (International Classification of Diseases) related to heart failure, along with the documented diagnosis in the electronic medical records, which provided detailed information on diagnoses, medications, and clinical outcomes. Eligible patients were 18 years or older, had a confirmed diagnosis of heart failure, and had been receiving guideline-directed medical therapy [16], including renin-angiotensin-system (RAS) inhibitors, beta-blockers, diuretics, and/or mineralocorticoid receptor antagonists, for at least three months. Sodium-glucose co-transporter 2 inhibitors (SGLT2i) were not commonly prescribed during the study period. All patients must have completed a one-year follow-up while on semaglutide.

Exclusion criteria included patients with NYHA class IV heart failure, a history of pancreatitis, known or suspected alcohol or drug abuse, uncontrolled thyroid disease, prior obesity treatments (including surgery, weight-loss devices, or medication), prior treatment with GLP-1 agonists, or significant renal impairment (eGFR < 15 mL/min/1.73 m²).

The decision to prescribe semaglutide was made independently by treating physicians as part of routine clinical care, based on their clinical judgment. Semaglutide was administered subcutaneously, starting at a dose of 0.25 mg per week, with the dose gradually escalated every four weeks, aiming to reach a maximum dose of 1.0 mg per week.

## Study endpoints

The primary endpoints of the study were changes in body mass index (BMI), improvements in NYHA class, and changes in the 6-minute walk distance (6MWD) from baseline to the one-year follow-up. Secondary endpoints included changes in NT-proBNP levels, mortality rates, and hospitalizations due to heart failure within the same time frame. The study received approval from the institutional review board (reference number 266/22) and was conducted in accordance with the Declaration of Helsinki and Good Clinical Practice guidelines.

### Statistical analysis

Patients in the semaglutide (SEMA) group were matched with those managed conservatively during the same time period using propensity scores (PS) obtained through multiple logistic regression. The variables used for the PS calculation included age, sex, body mass index (BMI), coronary artery disease, diabetes mellitus type 2, chronic obstructive pulmonary disease, estimated glomerular filtration rate (eGFR), New York Heart Association (NYHA) class (binary class I and II vs. III and IV), N-terminal pro-B-type natriuretic peptide (NT-proBNP), systolic blood pressure, heart rate, left ventricular ejection fraction (LVEF), and medication history, including Renin-angiotensin-system (RAS) inhibitors, Beta-blockers, diuretics, and Sodium-glucose co-transporter 2 (SGLT2) inhibitors. Using the nearest-neighbor algorithm based on the propensity scores, a one-to-one matching was performed.

Continuous variables were reported as mean ± standard deviation (SD) for normally distributed data or as median with interquartile range (IQR) for non-normally distributed data, with normality assessed by the Shapiro-Wilk test. Paired t-tests or Wilcoxon signed-rank tests were used for within-group comparisons, and unpaired t-tests or Mann-Whitney U tests for between-group comparisons, depending on the distribution. Categorical variables were presented as numbers with percentages and analyzed using the chi-square test. The Pearson correlation test was employed to examine the linearity of associations between variables. In addition, linear regression analyses were performed to further explore the predictive relationship between BMI changes and these clinical outcomes. For time-to-event analyses, p-values derived from a log-rank test were used for between-group comparisons. Hazard ratios (HR) and associated 95% confidence intervals (CI) for the treatment effects were estimated with the use of a Cox regression model. A two-sided p-value < 0.05 was considered statistically significant. Statistical analyses were performed using Stata/SE 17 (StataCorp, College Station, TX, USA).

## Results

### Study population

A total of 1942 patients were eligible for the study, with 57 patients receiving semaglutide (SEMA). After PS matching, 26 pairs of matched patients were identified. The mean duration of the one-year follow-up period was 12 ± 2 months.

In the unmatched cohort, patients receiving semaglutide exhibited significantly lower age, left ventricular ejection fraction (LVEF), and NT-proBNP levels, along with higher BMI, triglycerides, and a greater prevalence of diabetes mellitus type 2 compared to the unmatched control cohort (all *p* < 0.05). The matched cohort of patients receiving semaglutide was predominantly male (72%) with a mean age of 59 ± 12 years. Ischemic etiology was identified in 12 patients (46%). The mean LVEF was 41 ± 9%. The median level of NT-proBNP was 846 pg/ml (IQR: 328, 1592 pg/ml). Baseline characteristics are presented in Table 1. The distribution of propensity scores between both groups before and after PS matching is shown in **Supplemental Fig. 1**.

**Table 1:** Baseline characteristics

		Unmatched cohort			Matched cohort		
		SEMA	Control	P-value	SEMA	Control	P-value
No. of Patients		*n* = 57	*n* = 1885		*n* = 26	*n* = 26	
Age, yr.		53 ± 15	73 ± 12	< 0.001	59 ± 12	58 ± 19	0.86
Male sex		40 (70%)	1109 (59%)	0.10	20 (77%)	13 (50%)	0.83
BMI, kg/m²		37.1 ± 6.1	26.4 ± 6.9	< 0.001	34.0 ± 2.5	34.9 ± 3.9	0.32
Arterial hypertension		41 (72%)	1349 (72%)	1.00	21 (81%)	16 (62%)	0.22
Diabetes mellitus		25 (44%)	44 (24%)	0.001	14 (54%)	7 (27%)	0.09
Pulmonary disease		11 (19%)	489 (26%)	0.29	7 (27%)	7 (27%)	1.00
Coronary artery disease		25 (44%)	1017 (54%)	0.14	12 (46%)	8 (31%)	0.39
LVEF, %		40 ± 12	48 ± 24	0.02	41 ± 9	45 ± 15	0.20
	≤ 40%	26 (46%)	603 (32%)		13 (50%)	11 (42%)	
	41–49%	19 (34%)	339 (18%)		8 (31%)	7 (27%)	
	≥ 50%	11 (20%)	943 (50%)		5 (19%)	8 (31%)	
NYHA functional class				< 0.001			0.25
	II	33 (58%)	701 (38%)		15 (58%)	19 (73%)	
	III	12 (21%)	779 (42%)		11 (42%)	7 (27%)	
6MWD, m.		245 ± 183	229 ± 157	0.04	307 ± 134	285 ± 131	0.58
Serum creatinine, mg/dl		1.0 (0.9–1.3)	1.2 (1.0–1.6)	< 0.001	1.1 (0.9–1.4)	1.0 (0.9–1.3)	0.15
Triglycerides, mg/dl		166 (124–216)	105 (78–149)	< 0.001	167 (106–237)	122 (93–201)	0.28
HbA1c, %		6.1 (5.8–6.8)	5.9 (5.5–6.5)	0.06	5.8 (6.2–7.5)	5.8 (5.6–6.7)	0.12
NT-proBNP, pg/mL		439 (178–1015)	1837 (692–4081)	< 0.001	846 (328–1592)	909 (509–2263)	0.65
RAS inhibitor		54 (95%)	1346 (71%)	< 0.001	24 (92%)	20 (77%)	0.25
Beta blocker		55 (96%)	1478 (78%)	< 0.001	25 (96%)	21 (81%)	0.19
Diuretic		42 (74%)	1427 (76%)	1.00	26 (100%)	22 (85%)	0.11
SGLT2i		27 (47%)	356 (19%)	< 0.001	12 (46%)	8 (31%)	0.39

Values are either the number (%), mean ± SD, or median (interquartile range).

n.: number, yr.: years, BMI: body mass index, LVEF: left ventricular ejection fraction, NYHA: New York Heart Association, 6MWD: 6-minute walk distance, NT-proBNP: N-Terminal pro brain natriuretic peptide, RAS: Renin-angiotensin-system, SGLT2i: Sodium-glucose Cotransporter 2 Inhibitor.

### Dosage and side effects

The highest administered dose of semaglutide for any patient was 1.0 mg, with 0.5 mg being the maximum tolerated maintenance dose in four patients (15%). Nausea, experienced by six patients (23%), was the most common adverse reaction, leading to discontinuation of the medication in three patients (12%) after one year due to persistent symptoms. Additionally, one patient reported transient self-limited diarrhea. There were no reports of allergic reactions or symptomatic hypoglycemia.

## Weight changes

**Fig. 1:**
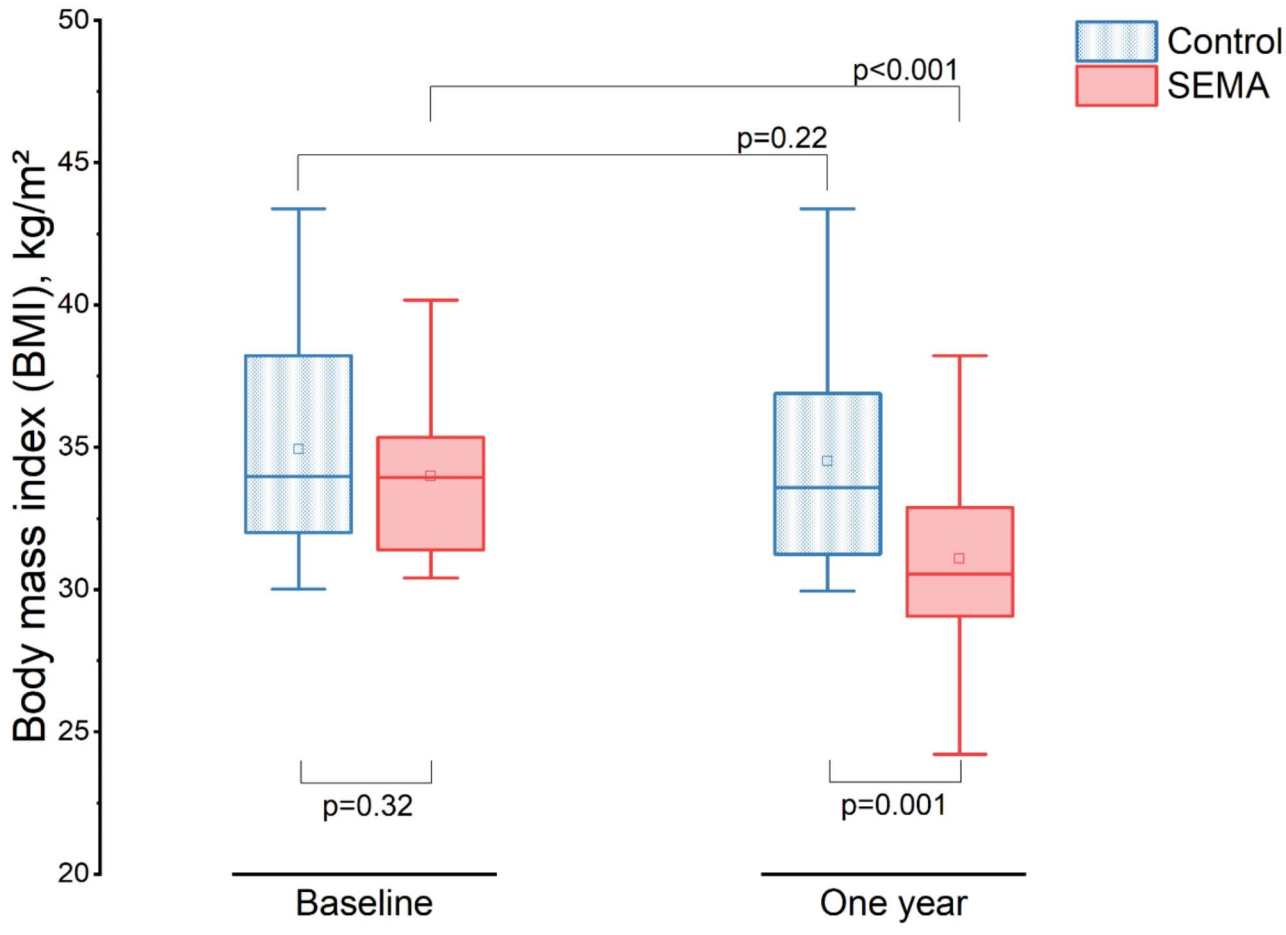
**Body mass index (BMI) at baseline and at one year for the matched SEMA and control groups** A significant reduction in BMI was observed in the SEMA group compared to the control group

At one year, significant weight loss from baseline was observed only in the SEMA group within the matched cohort, with a mean reduction of -8.88 kg (95% CI: -13.15 to -4.62; *p* < 0.001), compared to the control group (mean difference: -1.38 kg, 95% CI: -3.47 to 0.72; *p* = 0.19) **(Supplemental Fig. 2)**. In terms of BMI, the SEMA group showed a mean reduction of -2.91 kg/m² (95% CI: -4.27 to -1.55; *p* < 0.001), while the control group had a mean change of -0.41 kg/m² (95% CI: -1.08 to 0.26; *p* = 0.22) **(**Fig. 1**)**. The difference in BMI between the two groups at one year was statistically significant (mean difference: 3.42 kg/m², 95% CI: 1.43 to 5.42; *p* = 0.001).

## New York Heart Association (NYHA) functional classification

**Fig. 2:**
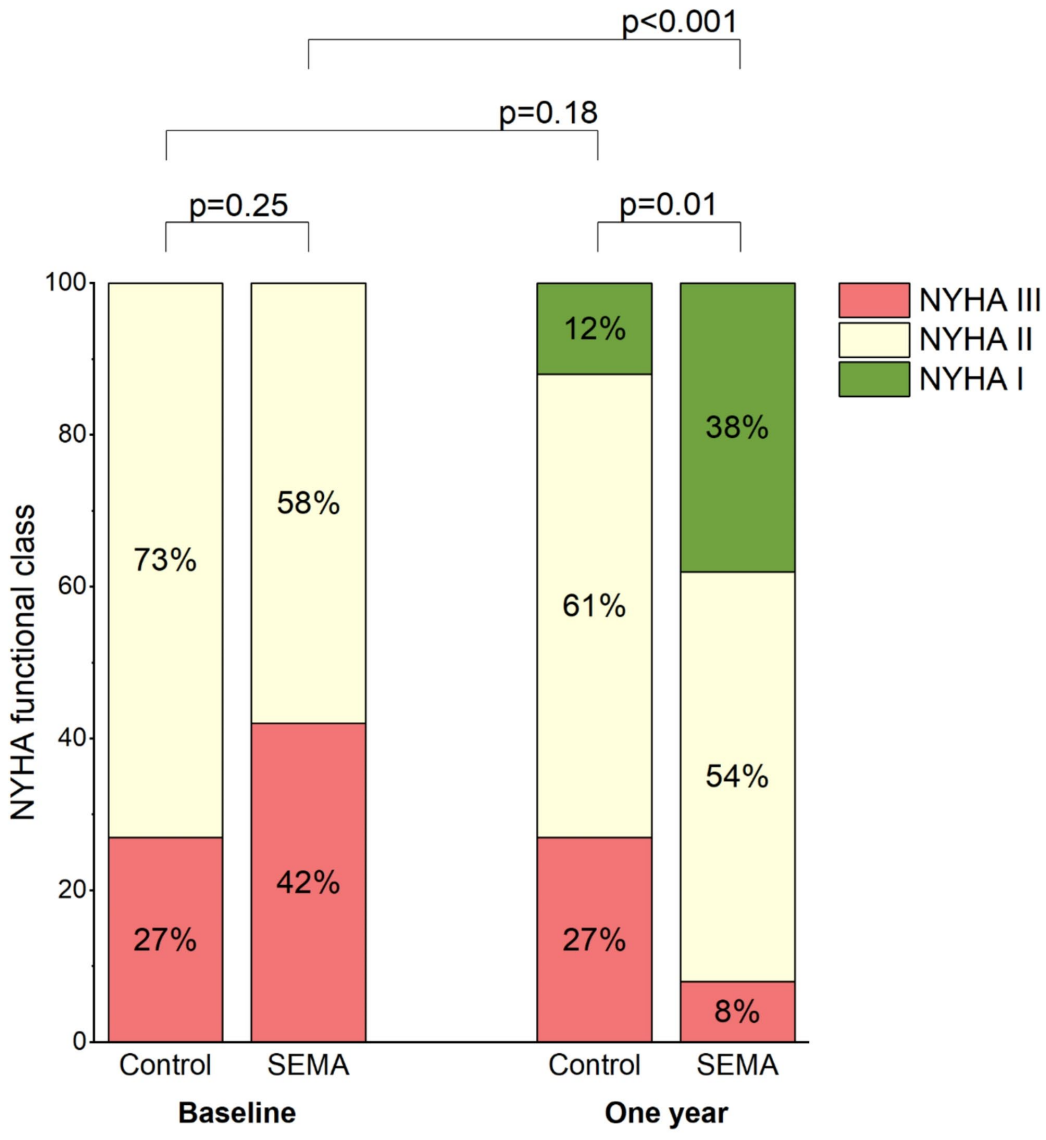
**New York Heart Association (NYHA) functional class at baseline and at one year for the matched SEMA and control groups** This figure highlights the improvement in NYHA functional class in the SEMA group compared to the control group

In the matched cohort, significant improvements in NYHA functional class from baseline were observed only in the SEMA group at one year, with 65% of patients showing improvement of at least one NYHA class (*p* < 0.001), compared to 15% in the control group (*p* = 0.18). Furthermore, patients on semaglutide more frequently achieved NYHA II or lower at the one-year follow-up compared to those in the control group (92% vs. 72%, *p* = 0.01) (Fig. 2).

### 6-minute walk distance (6MWD)

**Fig. 3:**
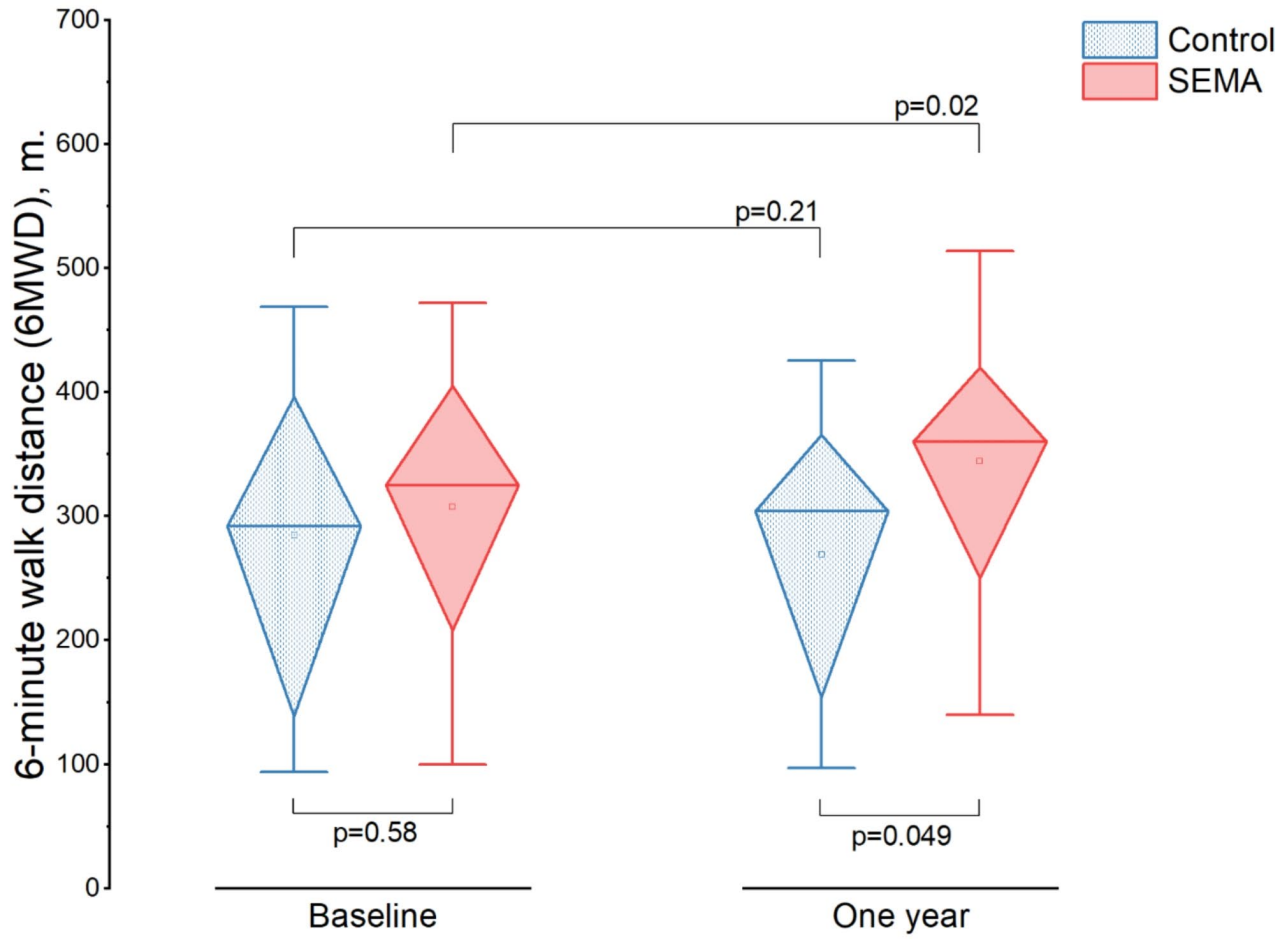
**6-minute walk distance (6MWD) at baseline and at one year for the matched SEMA and control groups** A significant increase in 6MWD was observed in the SEMA group compared to the control group

The SEMA group within the matched cohort demonstrated a significant improvement in 6MWD from baseline to one year, with a mean increase of 37 m (95% CI: 7.07 to 67.12; *p* = 0.02). In contrast, the control group had a non-significant mean change of -15 m (95% CI: -39.85 to 9.45; *p* = 0.21) **(**Fig. 3**)**. The difference in 6MWD between the two groups at one year was statistically significant, with a mean difference of 75 m (95% CI: 0.53 to 150.02; *p* = 0.049).

Additionally, within the SEMA group, changes in 6MWD were significantly negatively correlated with percentage changes in BMI (*r*= -0.64; *p* = 0.002), meaning that greater reductions in BMI were associated with greater improvements in 6MWD. Moreover, a linear regression analysis confirmed that the percentage change in BMI was a significant predictor of improvement in 6MWD (B = -4.37, 95% CI: -6.89 to -1.85; *p* = 0.002) **(Supplemental Fig. 3)**.

### N-terminal pro brain natriuretic peptide (NT-proBNP)

**Fig. 4:**
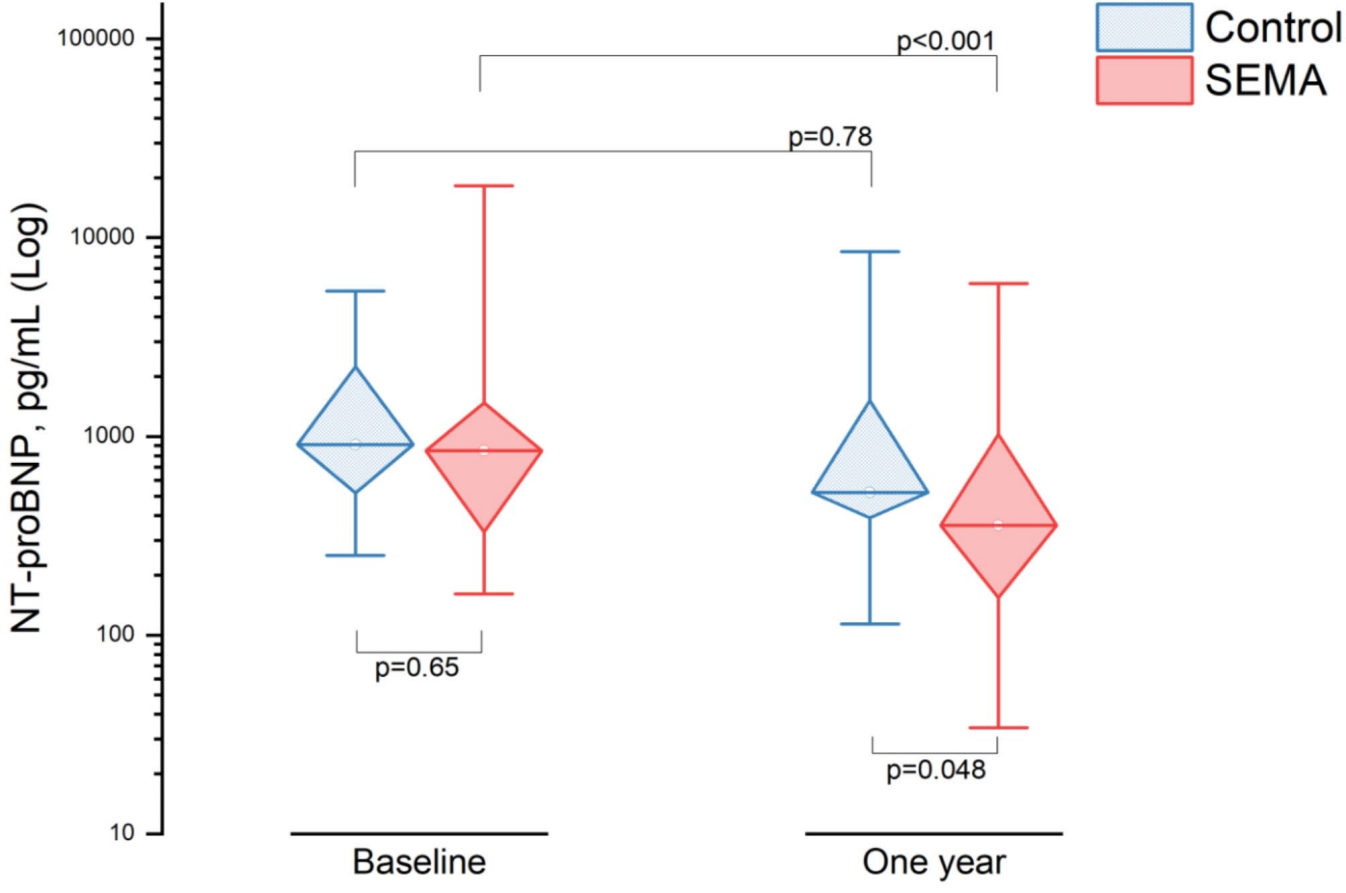
**NT-proBNP levels at baseline and at one year for the matched SEMA and control groups** A significant reduction in NT-proBNP levels was observed in the SEMA group compared to the control group

In the SEMA group within the matched cohort, NT-proBNP significantly decreased from a baseline of 847 pg/mL (IQR: 327, 1592 pg/mL) to 357 pg/mL (IQR: 152, 1055 pg/mL) at one year (*p* < 0.001, Fig. 4). In the control group, there were non-significant changes from 909 pg/mL (IQR: 509, 2264) at baseline to 522 pg/mL (IQR: 385, 1543) at one year (*p* = 0.78). The difference in NT-proBNP between the two groups at one year was statistically significant (*p* = 0.048). Changes in NT-proBNP levels in the SEMA group were significantly positively correlated with percentage changes in BMI (*r* = 0.52, *p* = 0.006). BMI percentage change was a significant predictor of improvement in NT-proBNP levels (B = 304, 95% CI: 94 to 514; *p* = 0.006) **(Supplemental Fig. 3)**.

### Left ventricular ejection fraction (LVEF)

In the matched cohort, there were no statistically significant differences in left ventricular ejection fraction (LVEF) between the SEMA and control groups. In the SEMA group, LVEF changed from 41 ± 9% at baseline to 44 ± 9% at the one-year follow-up (mean difference 2.86%, 95% CI: -0.22 to 5.94; *p* = 0.07). In the control group, LVEF changed from 46 ± 16% to 49 ± 13% during the same period (mean difference 3.21%, 95% CI: -1.35 to 7.78; *p* = 0.16, **Supplemental Fig. 3)**. In the semaglutide group, patients with LVEF < 50% decreased from 22 (85%) at baseline to 19 (73%; *p* = 0.83) in one year, with no significant difference. In the control group, the number dropped from 16 (62%) to 13 (50%; *p* = 0.83) in one year, also without significant difference.

### Mortality and heart failure-related hospitalizations

Within the one-year follow-up period, a total of four patients (16%) died, and nine patients (18%) were hospitalized due to heart failure within the matched cohort. The all-cause mortality rates were one patient (4%) in the SEMA group and three patients (12%) in the control group, with no significant difference between the two groups (HR: 2.89; 95% CI: 0.30 to 27.88; *p* = 0.36). Similarly, no significant differences were observed in terms of heart failure-related hospitalizations (HR: 0.91; 95% CI: 0.23 to 3.64; *p* = 0.89), with four patients (15%) in the SEMA group and five patients (19%) in the control group being hospitalized at least once due to heart failure.

## Discussion

In this study, we found that semaglutide is an effective weight-loss medication for obese patients with heart failure, associated with significant improvements in NYHA classification, 6-minute walk distance (6MWD), and NT-proBNP levels. However, promoting weight loss in this patient group remains a topic of ongoing debate. Research suggests that heart failure patients with higher BMIs often experience better outcomes than those with normal or low BMIs, a phenomenon known as the obesity paradox [17]. Nonetheless, this paradox could be due to BMI’s inability to differentiate between fat and muscle mass; lower BMI might reflect decreased muscle mass rather than reduced fat [18]. Skeletal muscle mass and function play a crucial role in cardiovascular outcomes, and their deterioration in chronic diseases is a strong predictor of a poor prognosis [19]. In advanced heart failure, there is a pathological shift toward a more catabolic state, leading to progressive muscle wasting, unintentional weight loss, and cardiac cachexia. These factors contribute to increased mortality, morbidity, and a decreased quality of life [20].

In this study, weight loss with semaglutide was associated with significant clinical and functional improvements. These findings add to the growing body of evidence challenging the obesity paradox and underscore the potential benefits of intentional weight reduction in heart failure patients, highlighting the value of weight management in this population [14, 15]. Therefore, it is important to differentiate between unintentional weight loss in advanced disease and intentional weight reduction aimed at improving physical function before reaching the terminal stages of heart failure.

Obesity is a common comorbidity in heart failure [21]. Adipose tissue exacerbates myocardial dysfunction by promoting inflammation, hypertension, dyslipidemia, and atherosclerosis [9, 22]. Additionally, it directly causes cardiac morphological, functional, and metabolic abnormalities, resulting in myocardial hypertrophy, stiffness, and fibrosis [9, 22]. With the increasing prevalence of both obesity and heart failure, there is an urgent need to explore novel pharmacologic options to address obesity in this patient population [8].

In recent years, semaglutide has emerged as a safe and effective weight-loss medication for obese adults, both with and without diabetes [11]. In the STEP-HFpEF trial, semaglutide demonstrated significant weight reduction in obese heart failure patients with preserved ejection fraction (HFpEF), achieving a mean loss of -8.4 kg compared to placebo after 52 weeks [15]. This is comparable to the mean weight reduction of -8.88 kg observed in our study, despite administering a maximum dose of only 1.0 mg. These findings emphasize the need for future studies to directly compare the efficacy, tolerance, and side effects of different dosing regimens in heart failure patients. Determining the optimal dose is particularly important in this cohort, as nausea was the most common side effect observed in our study, likely related to gut wall edema and compromised intestinal blood flow, both of which are commonly associated with heart failure [23].

Another key finding in our study was the significant improvements in NYHA functional class, 6MWD and NT-proBNP levels with semaglutide use. These results are consistent with subanalyses of the STEP-HFpEF trial in patients with heart failure and LVEF ≥ 45%[24, 25]. Whether the improvements in NT-proBNP or other clinical findings observed in our study are solely attributable to weight reduction or also due to the direct myocardial effects of semaglutide remains a topic of ongoing debate.

GLP-1 receptors (GLP-1Rs) have been identified not only in pancreatic beta cells but also in the myocardium and central nervous system, particularly within the nucleus tractus solitarius, a neuromodulatory center involved in cardiovascular control [26]. This suggests potential direct effects of GLP-1R agonists on myocardial structure and microvascular function [27, 28]. In the STEP-HFpEF trial, semaglutide use in obese heart failure patients with preserved LVEF was associated with a significant reduction in CRP levels, indicating a potential decrease in inflammation, a key factor in the development and progression of HFpEF [15]. These findings suggest potential effects for GLP-1R agonists beyond glycemic control and weight loss.

Obesity disrupts myocardial fatty acid oxidation, and weight loss has been shown to upregulate peroxisome proliferator-activated receptors (PPARs), potentially improving fatty acid metabolism and enhancing myocardial function [29]. Significant weight reduction, such as that observed after bariatric surgery, has been linked to reductions in left ventricular mass index and improvements in left ventricular function among obese heart failure patients [29, 30]. Studies on sodium-glucose cotransporter-2 inhibitors (SGLT2i) in patients with heart failure has also been associated with modest weight loss and significant reductions in heart failure-related hospitalizations [31, 32].

In the SELECT trial, semaglutide 2.4 mg used in patients with a BMI of ≥ 27 kg/m² and established cardiovascular disease without diabetes was associated with significant reductions in major adverse cardiovascular events (a composite of cardiovascular death, non-fatal myocardial infarction, or non-fatal stroke), as well as composite heart failure endpoints (cardiovascular death, hospitalization, or urgent hospital visit for heart failure) compared with placebo [14]. In a subanalysis of the STEP-HFpEF trial, semaglutide reduced the risk of worsening heart failure events in patients with HFpEF, though its effect on cardiovascular death alone was not significant [33]. Conversely, the GLP-1R agonist liraglutide in the LIVE trial was associated with more serious cardiac events in the treatment group than in the placebo group after 24 weeks [13]. Additionally, it did not result in significant changes to left ventricular systolic function compared with placebo in patients with stable chronic heart failure [13]. Similarly, in the FIGHT trial, liraglutide showed no significant difference compared to placebo in all-cause mortality or rehospitalization for heart failure among patients with LVEF ≤ 40% who were recently hospitalized for heart failure [12].

These contrasting findings highlight the variability in the effects of GLP-1 receptor agonists and underscore the need for further research to understand their differential impacts across diverse patient populations and clinical contexts. This research is essential to elucidate the relationship between obesity and cardiac function and to develop effective management strategies tailored to this specific patient group.

## Limitations

Our study provides valuable insights but also faces several limitations. Being a single-center study, the generalizability of the findings to a broader heart failure population is restricted. The relatively small sample size of 26 matched pairs limits the depth of data analysis. Moreover, the retrospective design of the study makes it susceptible to selection and information biases, despite our efforts to mitigate these through propensity score matching. Additionally, the maximum semaglutide dose used was 1.0 mg, lower than the 2.4 mg dose approved for weight loss, which may have constrained the observed effects on weight loss and clinical outcomes. The one-year follow-up period may also be insufficient to assess the long-term effects, safety, and potential adverse outcomes of semaglutide. Furthermore, our study did not extensively explore the mechanistic pathways through which semaglutide impacts heart failure, which could provide a deeper understanding of its benefits. Despite these limitations, the significant findings should inspire future research involving larger participant groups.

## Conclusion

Semaglutide induced significant weight reduction in obese patients with heart failure, accompanied by improved NYHA classification and 6-minute walk distance. Larger, multi-center trials and prospective, randomized controlled trials are warranted. These studies should focus on assessing long-term outcomes, optimizing dosage, and exploring the potential cardiovascular benefits beyond weight reduction.

## Perspectives

### What is known?

Obesity is a prevalent comorbidity in heart failure, and its incidence continues to increase. The potential clinical advantages of weight reduction in heart failure are yet to be explored, necessitating the identification of appropriate obesity drugs.

### What is new?

This study reveals that incorporating semaglutide into heart failure medical therapy is associated with a noteworthy decrease in body weight and substantial enhancements in clinical outcomes for obese patients with heart failure.

### What is next?

Future studies with larger participant groups are essential to validate these findings, paving the way for a more comprehensive understanding of the potential benefits of incorporating semaglutide in managing heart failure in individuals with excess weight.

## Electronic supplementary material

Below is the link to the electronic supplementary material.

Supplementary Material 1

## Data Availability

The datasets analyzed during the current study are not publicly available due to data protection requirements but are available from the corresponding author on reasonable request.

## Abbreviations

6MWD: 6-minute walking distance
AHA: American Heart Association
EF: ejection fraction
ESC: European Society of Cardiology
GLP-1R: glucagon-like peptide 1 receptor
IQR: interquartile range
LV: left ventricle/ventricular
m.: meter
NYHA: New York Heart Association
NT-proBNP: N-Terminal pro brain natriuretic peptide
PS: propensity score
