# Supplementary material for: Impact of semaglutide on weight and functional outcomes among obese heart failure patients: a propensity scores matching analysis
Source: BMC Cardiovasc Disord. 2024 Oct 26;24:590. doi: 10.1186/s12872-024-04275-2 (PMC11515153; doi:10.1186/s12872-024-04275-2)

**Supplemental materials**

**Supplemental Figure 1:** Distributions of propensity scores (PS) in both SEMA and control groups (A) before and (B) after matching.


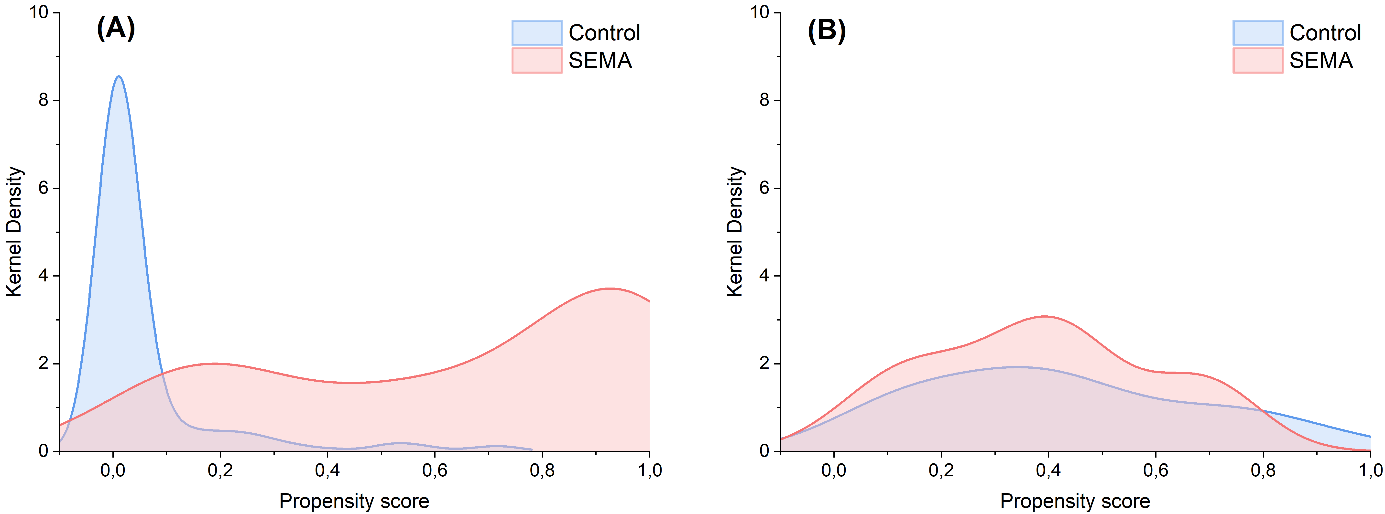


**Supplemental Figure 2: Distribution of percentage weight reduction in the SEMA and control groups at one year**

The SEMA group shows a significantly greater proportion of weight reduction compared to the control group.


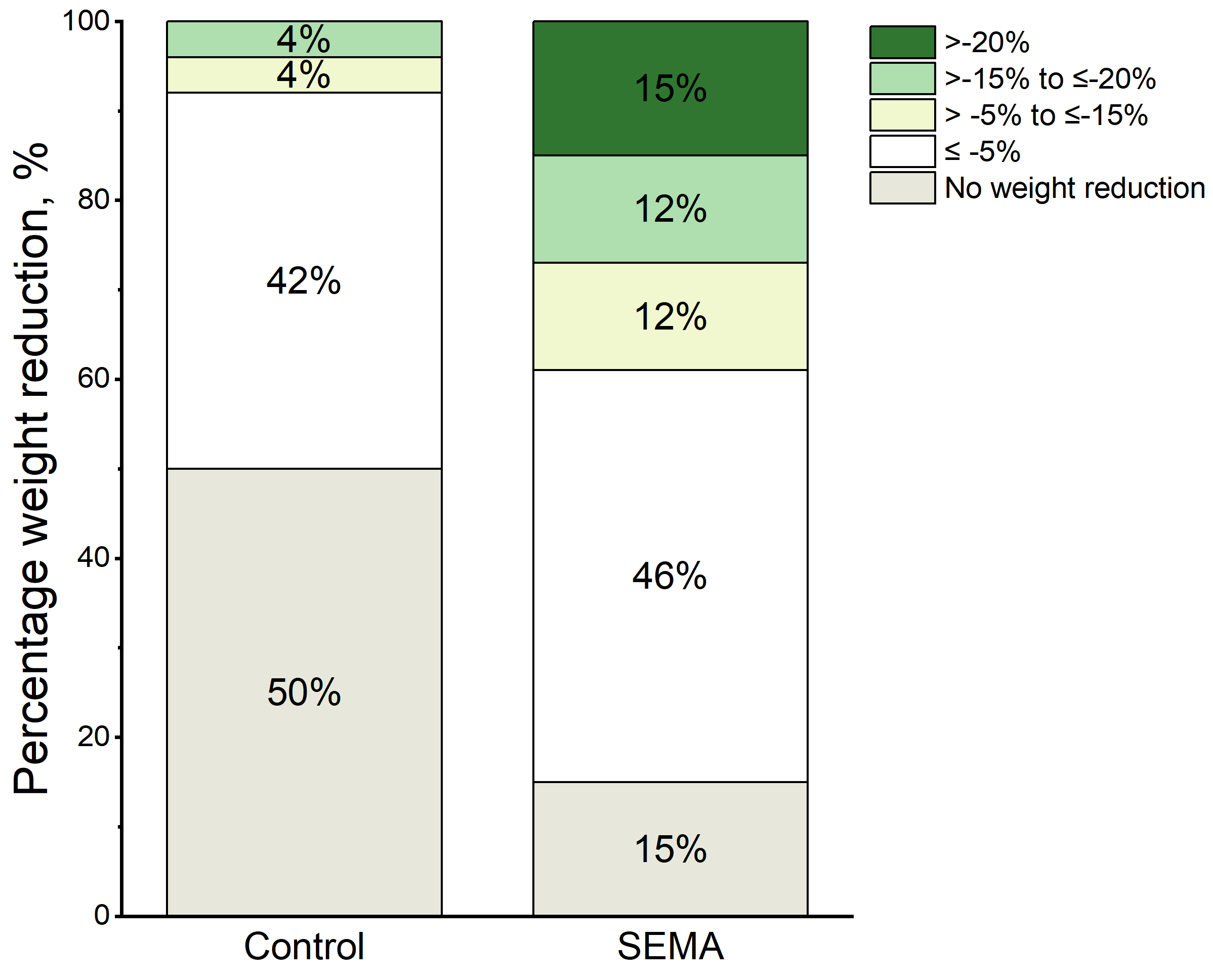


**Supplemental Figure 3: Correlation between percentage change of body mass index (BMI) and 6-minute walk distance (6MWD) change within the SEMA group from baseline to one year.**

There was a significant negative correlation, indicating that greater reductions in BMI in patients with heart failure are associated with greater improvements in 6MWD.


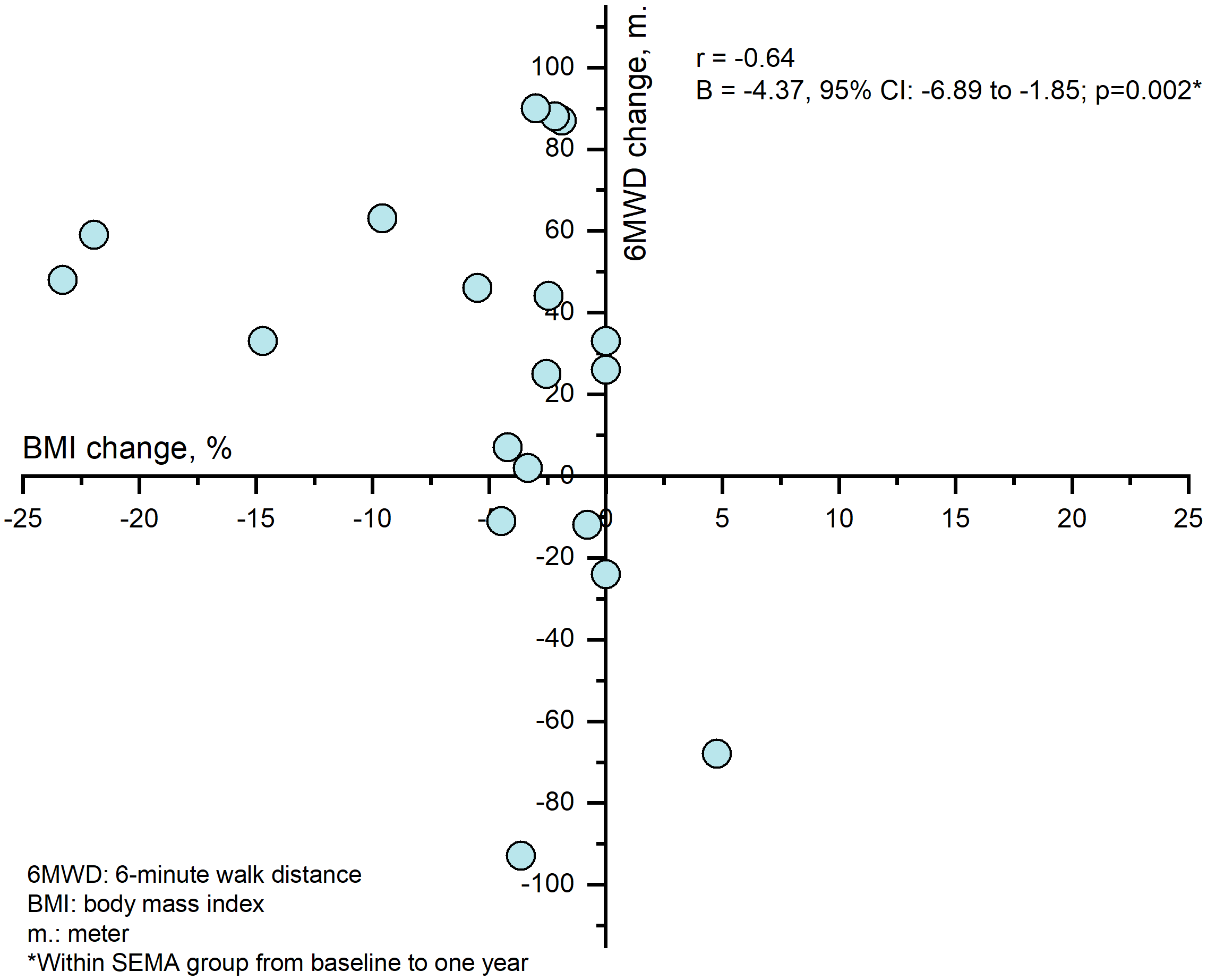


**Supplemental Figure 4: Correlation between percentage change of body mass index (BMI) and NT-proBNP change within the SEMA group from baseline to one year.**

There was a significant positive correlation, indicating that greater reductions in BMI were associated with greater reductions in NT-proBNP levels in patients with heart failure.


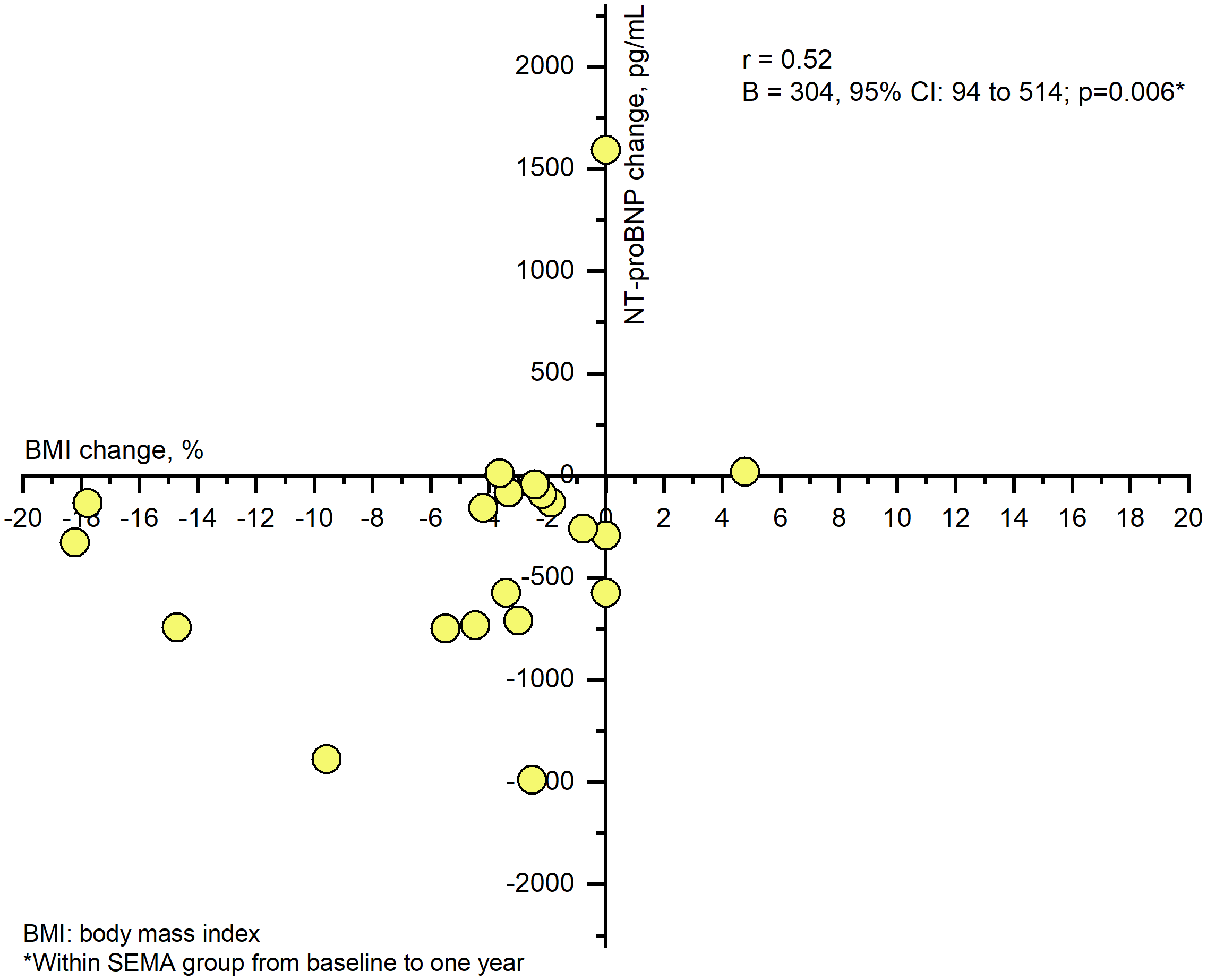


**Supplemental Figure 5: Left ventricular ejection fraction (LVEF) at baseline and one year for the SEMA and control groups.**

There was no statistically significant difference in LVEF between the SEMA and control groups at either baseline or one year.


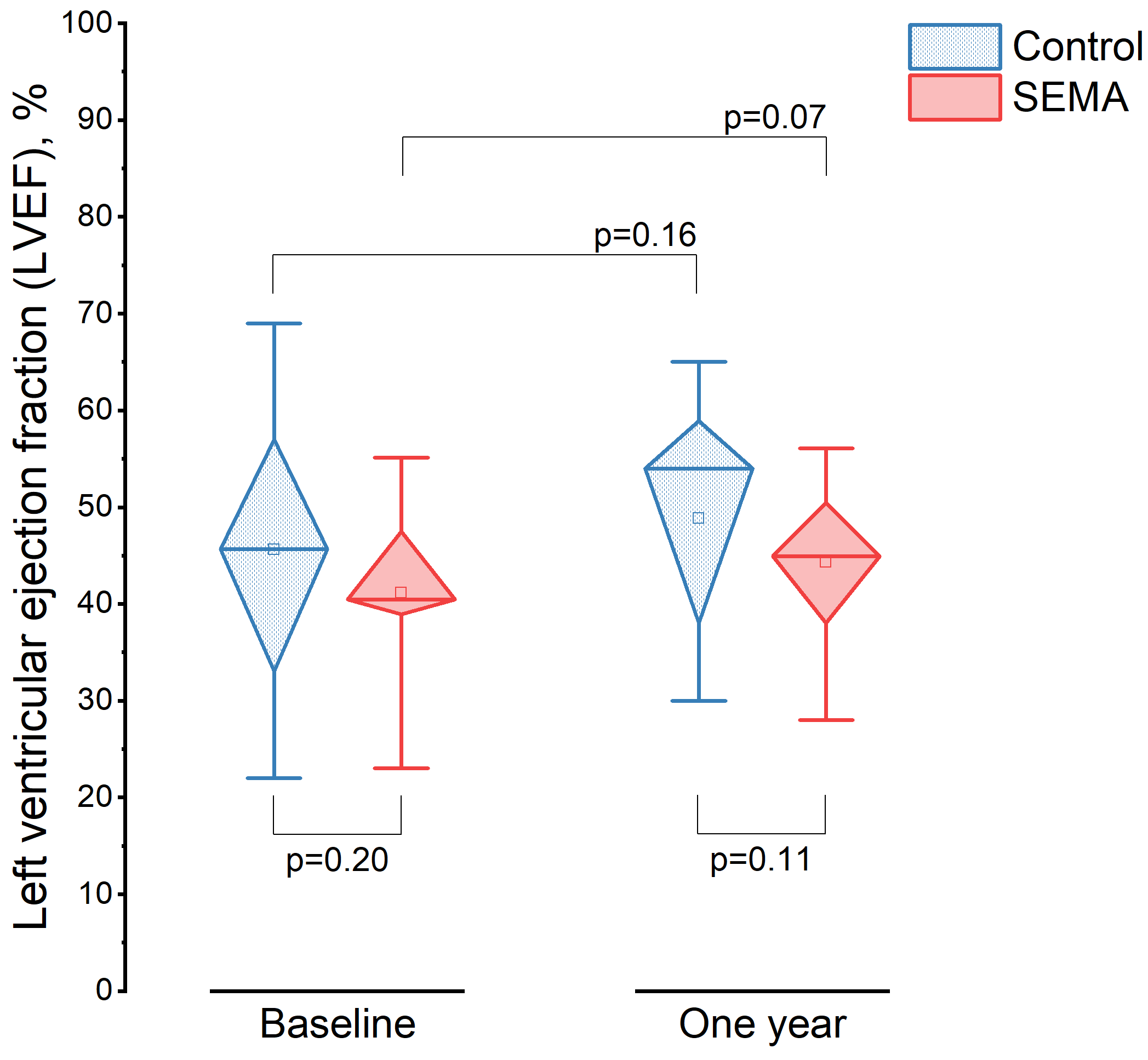

Supplement: Supplementary file 1 — Supplementary Material 1 [file 12872_2024_4275_MOESM1_ESM.docx]
